# Chronic *Toxoplasma* Infection Modifies the Structure and the Risk of Host Behavior

**DOI:** 10.1371/journal.pone.0032489

**Published:** 2012-03-14

**Authors:** Cristina Afonso, Vitor B. Paixão, Rui M. Costa

**Affiliations:** Champalimaud Neuroscience Programme, Instituto Gulbenkian de Ciência, Oeiras, Portugal; French National Centre for Scientific Research, France

## Abstract

The intracellular parasite *Toxoplasma* has an indirect life cycle, in which felids are the definitive host. It has been suggested that this parasite developed mechanisms for enhancing its transmission rate to felids by inducing behavioral modifications in the intermediate rodent host. For example, *Toxoplasma*-infected rodents display a reduction in the innate fear of predator odor. However, animals with *Toxoplasma* infection acquired in the wild are more often caught in traps, suggesting that there are manipulations of intermediate host behavior beyond those that increase predation by felids. We investigated the behavioral modifications of *Toxoplasma*-infected mice in environments with exposed *versus* non-exposed areas, and found that chronically infected mice with brain cysts display a plethora of behavioral alterations. Using principal component analysis, we discovered that most of the behavioral differences observed in cyst-containing animals reflected changes in the microstructure of exploratory behavior and risk/unconditioned fear. We next examined whether these behavioral changes were related to the presence and distribution of parasitic cysts in the brain of chronically infected mice. We found no strong cyst tropism for any particular brain area but found that the distribution of *Toxoplasma* cysts in the brain of infected animals was not random, and that particular combinations of cyst localizations changed risk/unconditioned fear in the host. These results suggest that brain cysts in animals chronically infected with *Toxoplasma* alter the fine structure of exploratory behavior and risk/unconditioned fear, which may result in greater capture probability of infected rodents. These data also raise the possibility that selective pressures acted on *Toxoplasma* to broaden its transmission between intermediate predator hosts, in addition to felid definitive hosts.

## Introduction

*Toxoplasma gondii* (from here on referred to as *Toxoplasma*) is an obligate intracellular parasite [1], capable of infecting all mammals, with an indirect life cycle where cats and other felids constitute the definitive hosts. In infected felids, parasites will invade the epithelial cells of the intestine, where they undergo both sexual and asexual reproduction. Felids are the only mammals known to shed *Toxoplasma* oocysts with their feces [2], which will contaminate the surrounding environment. When oocysts are ingested by a mammal other than a cat, such as a wild rodent (intermediate host), an extra-intestinal cycle is initiated, where asexual (clonal) reproduction occurs and small cysts form in various tissues, most notably in the brain. In the intermediate host, infection will persist in a chronic, latent state. If a non-infected cat then consumes an infected intermediate host, the *Toxoplasma* life cycle is completed [3].

Given that sexual reproduction occurs exclusively in felids, it can be argued that this parasite would be under strong selective pressure to develop mechanisms to increase its transmission from the intermediate to the definitive host. This increase in transmission rate could be related to the presence of parasitic cysts in the brain of intermediate rodent hosts. Several studies have shown that *Toxoplasma*-infected rodents exhibit a number of modifications in their behavior [4], [5], [6], [7] and, most notably, that they display an altered response to feline predator odor, with loss of the typical aversion and even the development of attraction [8], [9], [10]. This attraction/loss of aversion to cat odor has been suggested as a mechanism through which *Toxoplasma* enhances its transmission from the intermediate to the definitive host. However, the fact that wild rodents with naturally acquired *Toxoplasma* infection are more frequently caught in traps [11] argues for additional manipulations of the behavior of the intermediate host, which could increase capture in general and not only capture by felids. The aims of this study were to investigate the existence of behavioral modifications that could lead to increase capture probability of *Toxoplasma*-infected rodents, and to determine whether these behavioral modifications were correlated with specific cyst localizations in the brain.

To address these questions, we investigated the behavior of mice chronically infected with *Toxoplasma* in two different environments, each with exposed areas that animals typically avoid and non-exposed areas that animals prefer. We found that chronically infected mice with brain cysts display a plethora of behavioral alterations in these environments. We then used principal component analysis to identify, in an unsupervised manner, the main factors that explain the behavioral changes observed. Finally, we analyzed cyst distribution across the whole brain of infected animals and investigated whether there was a correlation between the behavioral changes and cyst distribution and localization.

Our results demonstrate that chronic infection by *Toxoplasma* changes the microstructure of exploratory behaviors and risk/unconditioned fear, and that these changes are related to cyst presence and cyst localization. These data could explain the increased capture probability reported for infected rodents in the wild, and raise the possibility of selective pressures acting on *Toxoplasma* to increase transmission not only to its feline definitive host, but also to predator hosts in general.

## Results

### Animals with brain cysts display alterations in weight gain during the acute stage of infection

**Figure 1:**
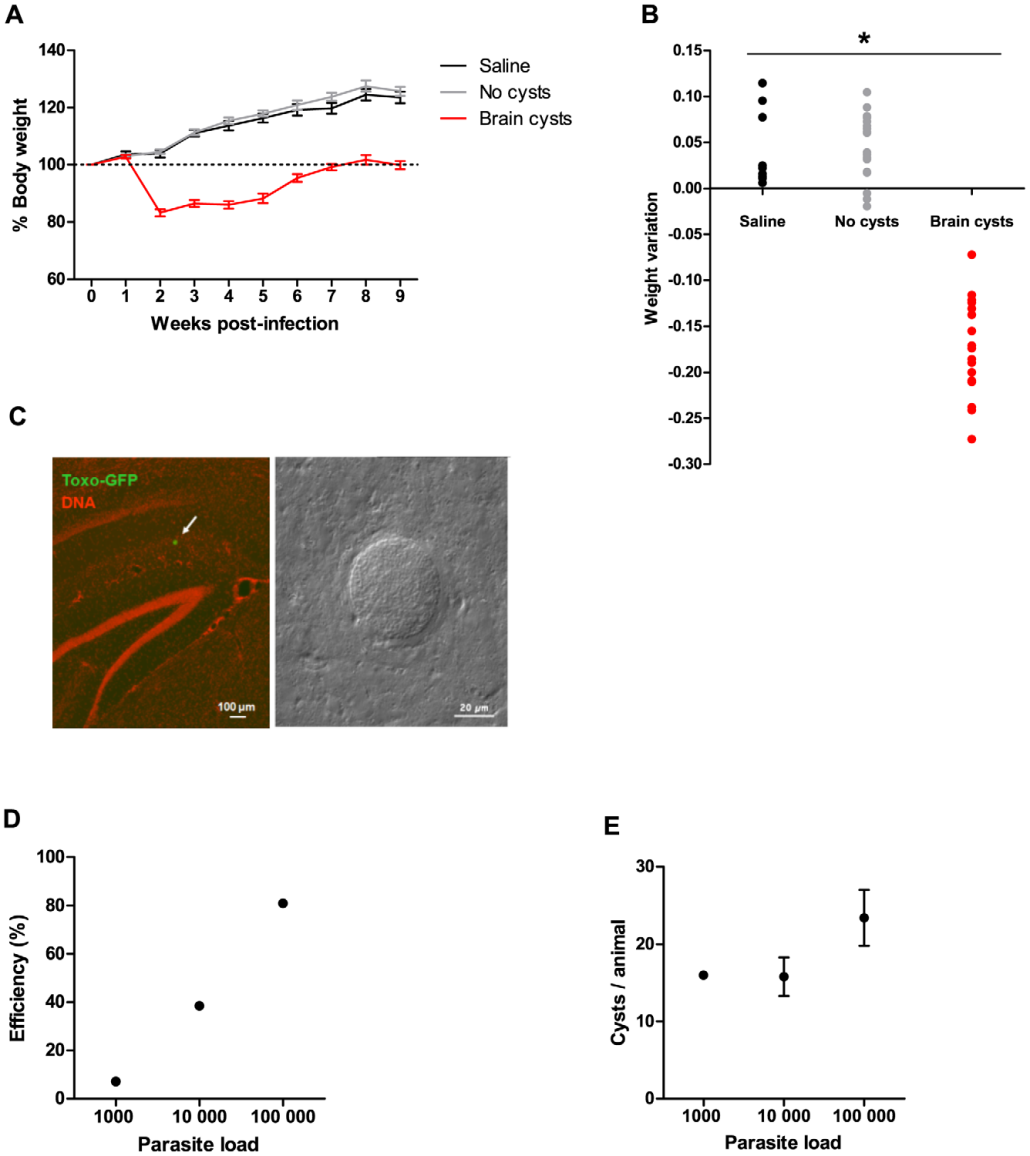
Dynamics of *Toxoplasma* infection in C57BL/6J mice. (A) Weight variation during infection. Acute infection resulted in severe weight loss in the first two weeks, followed by a recovery phase, with animals returning to initial weight values nine weeks post-injection (dotted line). (B) All animals with acute weight loss (>5%) during the first two weeks post-infection developed brain cysts. (A,B) Saline group, n = 10; No cysts group, n = 20; Brain cysts group, n = 19. (C) Immunofluorescence and phase contrast images of GFP-expressing cysts in the mouse brain. DNA was artificially stained in red. (D) Efficiency of chronic infection increased with number of parasites injected (injected animals: 1000 parasites n = 14; 10 000 parasites n = 13; 100 000 parasites n = 21). (E) Average number of cysts formed in the mouse brain did not depend on the number of parasites injected (1000 parasites n = 1 out of 14; 10 000 parasites n = 5 out of 13; 100 000 parasites n = 17 out of 21).

We injected C57BL/6J female mice intraperitoneally with transgenic *Toxoplasma gondii* ME49 tachyzoites, previously shown to display infection dynamics similar to the parental strain [12], and monitored them for 9 weeks (see Materials and Methods). Infected animals that developed brain cysts (“Brain cysts” group) went through an acute stage of infection characterized by weight loss (Figure 1A, *F*_(7.70,176) = _47.5, p<0.05), followed by a recovery stage that led to the chronic phase of the disease. Seven weeks after parasite injection, animals with brain cysts had returned to the initial weight (dotted line in Figure 1A). In control animals injected with saline (“Saline” group), as well as animals injected with parasites but that did not develop brain cysts (“No-cysts” group), weight loss was not observed. Moreover, weight loss in the first two weeks after injection was predictive of brain cyst development, since brain cysts were found exclusively in infected animals with more than 5% weight loss (Figure 1B, *F*_(2,46)_ = 138, p<0.05). Accordingly, animals that developed brain cysts (Figure 1C) tested positive for the presence of anti-*Toxoplasma* antibodies in a latex particle agglutination test using blood serum. However, all animals in the No-cysts group tested negatively for the presence of antibodies, similarly to what was observed in the Saline group (data not shown). Therefore, animals in the Saline and No-cysts groups were considered as controls for injection and infection, respectively.

The infection efficiency varied according to parasite load injected, with higher parasite loads resulting in a higher proportion of cyst-containing animals (Figure 1D). However, the number of brain cysts formed was not dependent on the injected parasite load (Figure 1E, *H*_(2)_ = 2.72, ns, p = 0.26; average of 22.3±2.70 cysts/brain), similar to what has been previously reported for lower *inoculum* sizes [6], [13]. Taken together, these data showed that chronic *Toxoplasma* infection resulted in the formation of parasite cysts in the brain, which could be predicted by the loss of weight during the acute phase of infection.

### Chronic *Toxoplasma* infection alters exploratory locomotion

To determine whether there were general alterations in exploratory behavior in chronically infected animals (9 weeks post-infection), we analyzed their performance in the open field test (reviewed in [14]), consisting of a novel arena with more exposed areas (center of the arena) and less exposed areas (border of the arena). Each animal was placed at the center of the square arena, and spontaneous behavior was measured for 10 min (Video S1 and Video S2).

**Figure 2:**
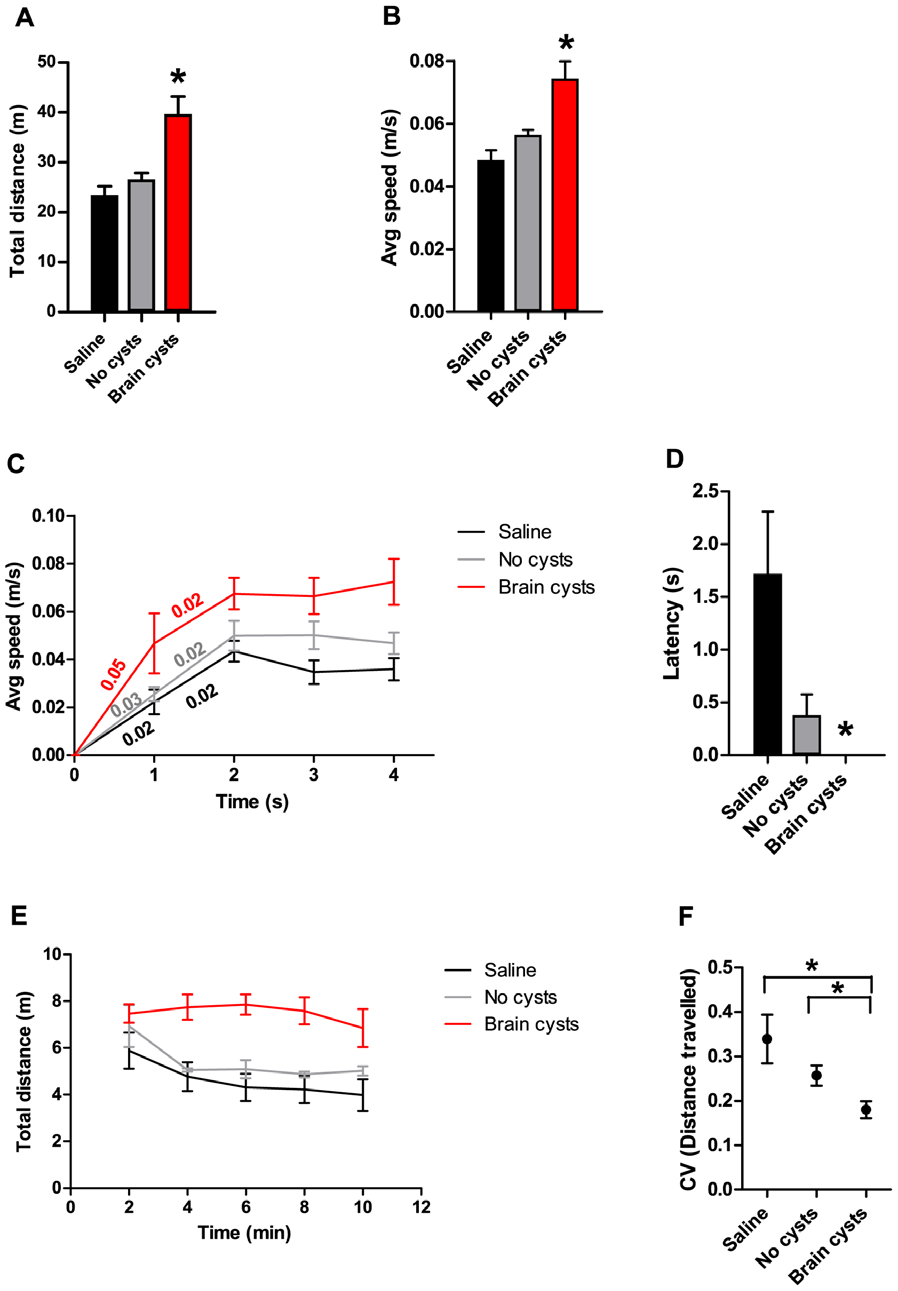
Chronically infected mice display alterations in exploratory behavior in the open field. (A) Compared to control animals, infected mice showed an increase in the total distance travelled in the arena. (B) Animals that contain brain cysts moved at higher speeds in the open field than controls. (C) The initial acceleration during a movement bout was higher in animals with brain cysts. Centesimal values indicate line slopes in one second intervals. (D) Infected animals showed no latency to move after being placed in the open field, in contrast to control groups. (E) In contrast to what was observed in control animals, no habituation to the novel environment was observed in infected animals, as shown by the absence of the reduction in locomotion over time. (F) The distance covered was more uniform over time in cyst-containing animals, as indicated by the reduced coefficient of variation when compared to control groups. (A–F) Saline group, n = 10; No cysts group, n = 20; Brain cysts group, n = 19.

Animals with brain cysts showed an increase in the total distance travelled in the arena when compared with control animals (Figure 2A, *Fw*R_(2, 25.5)_R = 6.95, p<0.05). Furthermore, the animals with cysts moved at higher average speed within the arena, relative to control groups (Figure 2B, *H*R_(2)_R = 13.7, corrected for multiple comparisons p<0.02, maximum speed was not different across groups). Animals with brain cysts reached higher locomotion speeds as early as the first second of a locomotion segment (Figure 2C, *F*R_(2, 46)_R = 9.49, p<0.05), and showed an initial acceleration during that first second of movement which is double that of control groups.

As expected, animals in the injection control group (Saline) displayed a latency to move (average of 1.71±0.59 s) immediately after being placed in the center of the arena (Figure 2D, Video S3 and Video S4), which is the characteristic response during the first contact with an exposed area in a novel environment [15]. Interestingly, this latency period was reduced in No-cysts animals (0.37±0.20 s), and absent in all of the cyst-containing mice (Figure 2D, 0.0±0.0 s, *H*R_(2)_R = 12.8, corrected for multiple comparisons p<0.02).

Animals from both control groups habituated to the test environment, as indicated by the decline in distance covered, in blocks of 2 minutes, over the 10 minute test. However, animals with brain cysts did not habituate (Figure 2E, main effect of treatment *FR*_(2,46)_R = 8.86, p<0.05, *post hoc* tests significant between treatment groups for mins 4, 6, 8, 10). This was confirmed when analyzing the coefficient of variation for each animal over time: animals with brain cysts showed significantly less minute-to-minute variability when compared to control groups (Figure 2F, *Fw*_R(2,20.9)R_ = 5.62, p<0.05).

Overall, the results described above show that animals with chronic *Toxoplasma* infection displayed more exploratory locomotion, with higher locomoting speed and acceleration. They also show decreased latency to first move in a novel environment and different habituation to that environment.

### Micro-structure of exploratory locomotion is affected in chronically infected animals

**Figure 3:**
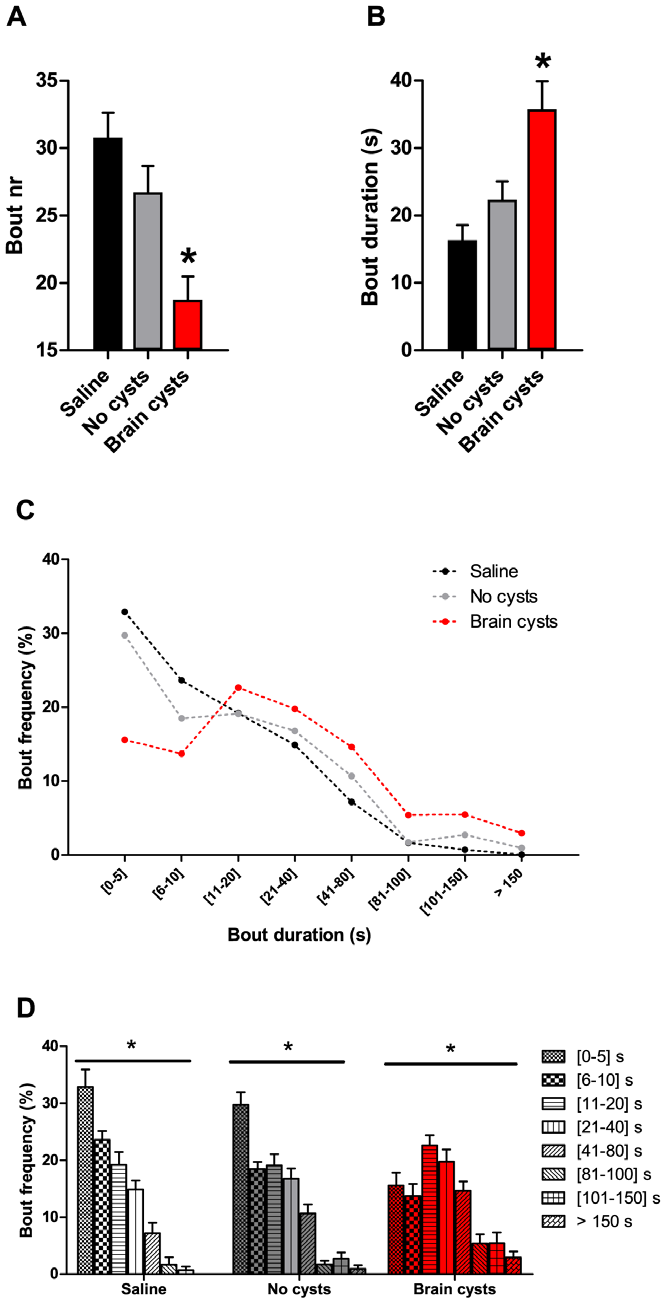
*Toxoplasma*-infected mice show changes in the structure of exploratory movement. (A) Movement bouts were reduced in animals with brain cysts, relative to control groups. (B) Chronically infected animals moved for longer periods at a time, as evidenced by the increased bout duration. (C,D) Movement structure was changed in cyst-containing mice compared with controls, with a reduction in the number of shorter bouts, and an increase in the frequency of long and very long bouts. (A–D) Saline group, n = 10; No cysts group, n = 20; Brain cysts group, n = 19.

We observed an overall increase in distance travelled and average locomotion speed in mice with brain cysts. However, these effects could result from animals moving more or faster, or from changes in the structure of their locomotion. We found that these changes reflected a disruption of the way animals organized their exploratory movements. Detailed analysis of movement segments or bouts (Figure 3, see Materials and Methods) showed that, when compared to control groups, locomotion of cyst-containing mice was characterized by a reduction in the number of movement bouts (Figure 3A, *F*R_(2,45)_R = 9.18, p<0.05) and an increase in their duration (Figure 3B, *H*R_(2)_R = 14.8, corrected for multiple comparisons p<0.02).

Further analysis of the distribution of bout duration frequencies showed that control groups exhibited similar movement structure, with shorter bouts occurring more frequently than longer bouts (Figure 3C,D). This structure was altered in animals containing brain cysts, with a marked decrease in the number of short bouts, an increase in the frequencies of intermediate and long duration bouts, and the appearance of very long bouts, which were absent in the control groups (Figure 3D, Saline *F*R_(3.07, 27.6)_R = 41.8; No-cysts *F*R_(3.60, 68.3)_R = 40.8; Brain cysts *F*R_(4.10, 73.8)_R = 13.2, p<0.05). *Post hoc* tests showed that, in control groups, short bouts ([0–5] s) outnumbered bouts of intermediate duration ([21–40] s) while in cyst-containing animals no such difference was found.

These results demonstrated that chronic *Toxoplasma* infection drastically modifies the organization and the structure of exploratory locomotion in mice. Infected animals moved uninterruptedly for longer periods of time in a novel environment with exposed areas.

### Infected animals display differential behavior in exposed *versus* non-exposed areas

**Figure 4:**
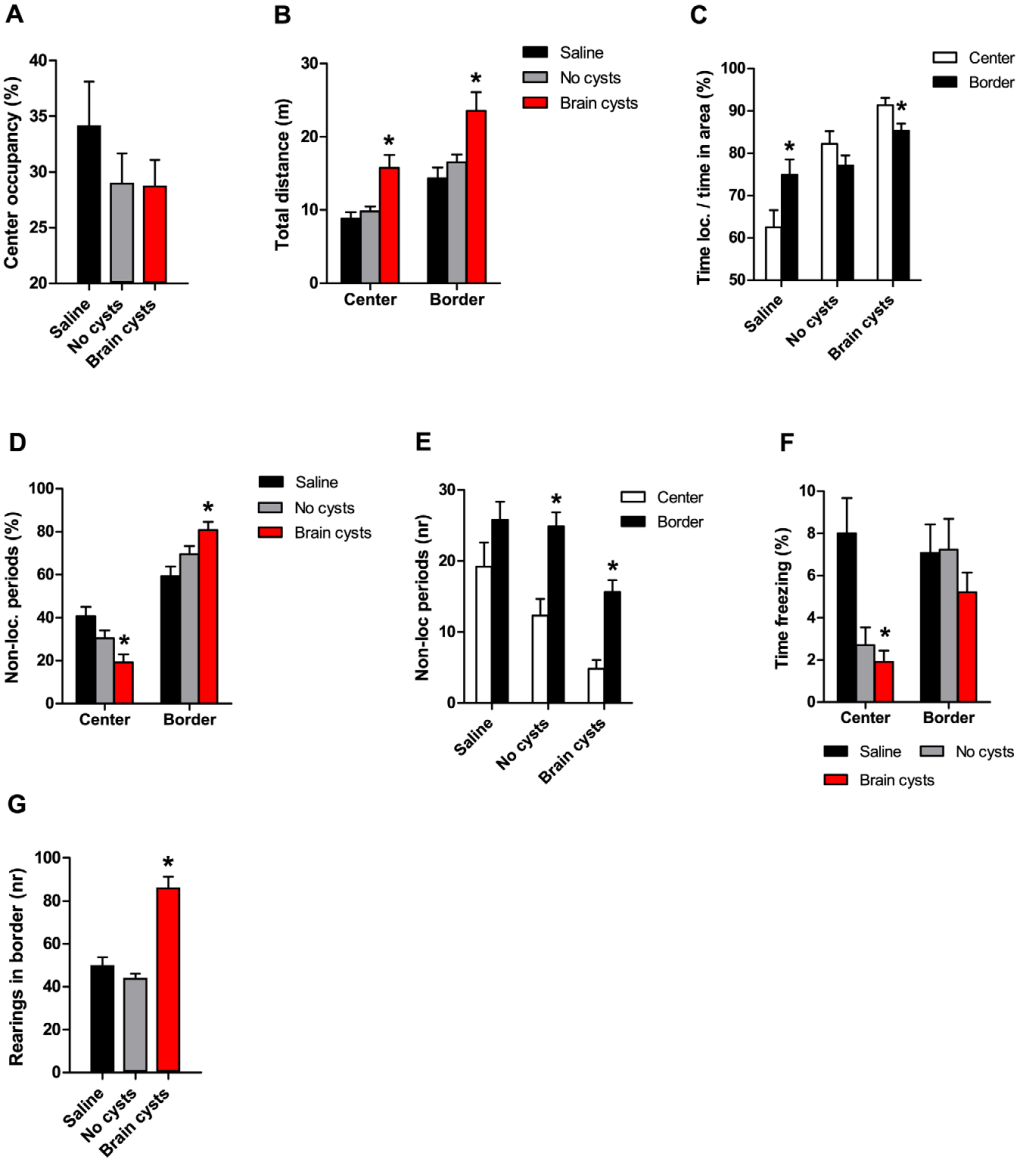
Different behavioral alterations in exposed *versus* non-exposed areas of the open field. (A) Infected animals did not show an altered preference for center area occupancy. (B) Animals with brain cysts displayed increased locomotion in both zones of the arena when compared with control groups. (C) In contrast to Saline animals, cyst-containing mice engaged in locomotion preferentially in the center zone. (D,E) Chronically infected animals showed a smaller number and percentage of non-locomoting periods in the center zone (F) Infected animals displayed a reduction in percent time freezing exclusively in the center zone. (G) The number of rearings against the arena walls was higher in animals with brain cysts than in control groups. (A–G) Saline group, n = 10; No cysts group, n = 20; Brain cysts group, n = 19.

The longer bouts of movement described above could reflect periods of uninterrupted movement spanning both the center and border zones of the open field. We therefore analyzed in more detail the structure of behavior in the center and border zones. Mice will normally avoid the more exposed area (center zone) in favor of the protected one (border zone, close to arena walls, reviewed in [16], [17]). We found that, for all groups, animals spent more time in the border area, with no significant differences in center occupancy (Figure 4A, *F*R_(2,46)_R = 0.83, ns). Furthermore, chronically infected animals showed increased locomotion both in the center (Figure 4B, *Fw*R_(2,25.3)_R = 6.13, p<0.05) and border zones (Fig. 4B, *Fw*R_(2,25)_R = 4.98, p<0.05) of the arena. We then investigated if the different experimental groups showed distinct patterns of behavior during the time spent in the different zones of the open field. In fact, Saline control mice spent less time engaged in locomotion in the center when compared with the border zone (Figure 4C, t(9) = −5.40, p<0.05), while no such difference was found in the No-cysts group. In contrast, cyst-containing animals showed a reversal of this preference, spending more time engaged in locomotion in the center when compared to the border zone (Figure 4C, t(18) = 4.50, p<0.05).

In agreement with the results above, we found that, in the center zone, the relative number of non-locomoting periods (i.e. periods without locomotion or horizontal movement, including grooming, rearing, freezing, etc) was significantly reduced in chronically infected animals with brain cysts relative to Saline animals (Figure 4D, *H*R_(2)_R = 11.0, corrected for multiple comparisons p<0.02, with corresponding increase in the border area). Also, while there was no difference in the absolute number of non-locomoting periods between center and border zones in Saline animals (Figure 4E, t(9) = −1.91, ns, p = 0.09), a clear reduction in the number of non-locomoting periods in the center was apparent in both No-cysts and cyst-containing mice (Figure 4E, No-cysts, t(19) = −4.18; Brain cysts, t(18) = −6.58, p<0.05). Regarding time spent freezing in each zone (see Materials and Methods), we verified that cyst-containing mice and No-cysts animals showed almost no freezing in the center zone, as opposed to saline controls (Figure 4F, *H*R_(2)_R = 11.6, corrected for multiple comparisons p<0.02). However, when measuring behavior in the border zone, cyst-containing animals showed a higher number of rearings against the wall than control groups (Figure 4G, *F*R_(2,46)_R = 31.6, p<0.05).

These results revealed that even though chronically infected animals showed preference for non-exposed rather than exposed zones in terms of relative occupancy, they displayed very different behaviors when in these areas. While in the center area, cyst-containing mice were mostly locomoting, with very few periods of freezing or other non-locomoting episodes, possibly reflecting differences in terms of the ability to assess risk in an exposed area. Interestingly, many of the same patterns were observed in the No-cysts group, suggesting that these behaviors are triggered by the contact with the parasite and not by chronic infection with formation of brain cysts. However, cyst-containing animals did perform more rearings against the wall in the border zone than either of the control groups.

### Fear/risk-related responses are abnormal in chronically *Toxoplasma*-infected animals

**Figure 5:**
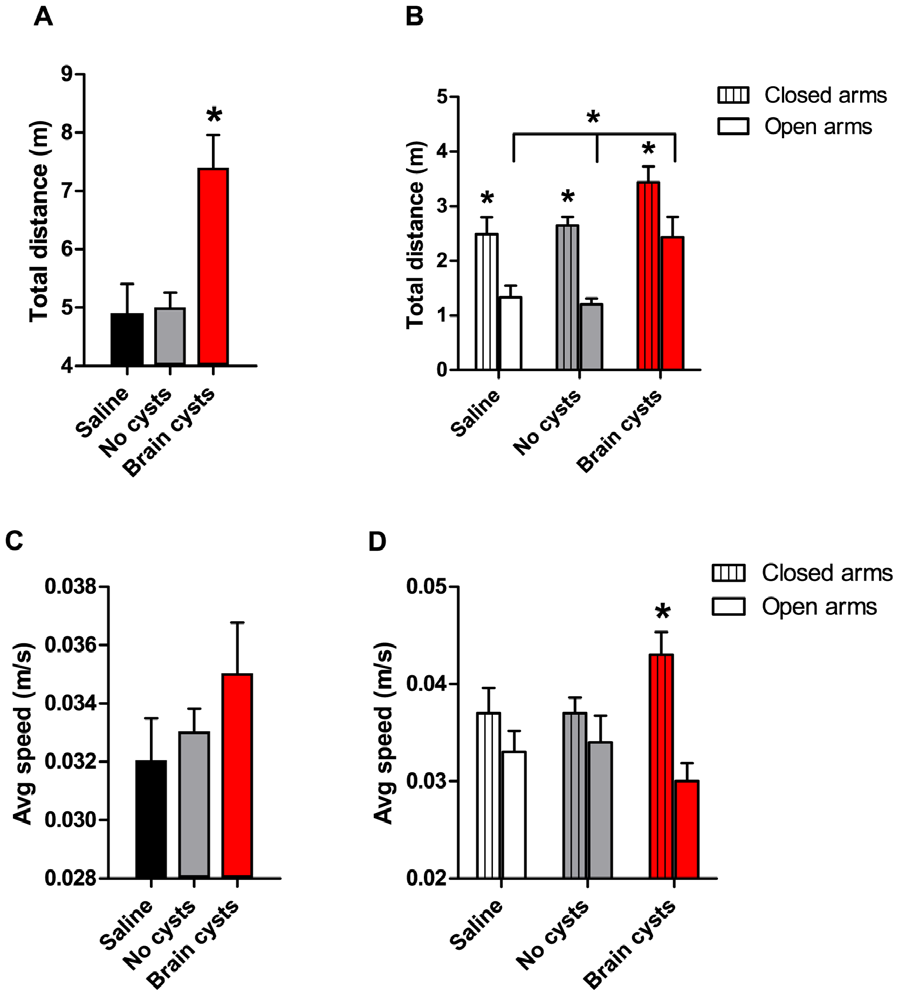
Altered behavior in the elevated plus maze in mice with chronic *Toxoplasma* infection. (A) Infected animals exhibited an increase in the distance travelled in the apparatus relative to controls. (B) Cyst-containing animals covered longer distances than control groups in the open arms. (C) Overall average speed remained unaffected across all experimental groups. (D) In contrast to control groups, infected animals displayed higher speed in closed arms than in open arms. (A–D) Saline group, n = 10; No cysts group, n = 20; Brain cysts group, n = 19.

The behavioral alterations reported above in animals with brain cysts suggested that these animals behaved differently in exposed *versus* non-exposed areas when compared to controls. To further characterize this behavior, we used the elevated plus maze test which can reliably assess unconditioned fear-related responses in rodents (reviewed in [18]). This apparatus consisted of four arms (two open and two closed) arranged in a plus shape and elevated from the floor. The test relies upon the innate preference for dark, enclosed spaces and the unconditioned fear of heights/open spaces exhibited by mice; closed arms constitute safe zones whereas open arms represent unsafe areas. Consistent with the results obtained in the open field, animals with brain cysts showed an increase in the overall distance travelled during the 5 min test (Figure 5A, *F*_(2,45)_ = 9.18, p<0.05), when compared to control groups. Further analysis revealed that this difference in locomotion between groups emerged primarily because animals with brain cysts displayed increased locomotion in open arms compared to controls (Figure 5B, *Fw*_(2,21.8)_ = 5.08, p<0.05, *post hoc* tests for cyst-containing mice *versus* controls, p<0.05; Video S5 and Video S6). Moreover, even though no striking differences were detected in overall speed between groups (Figure 5C, p>0.05), cyst-containing animals moved faster in the closed arms than in the open arms (Figure 5D, *F*_(2,45)_ = 3.56, p<0.05, *post hoc* tests p<0.05).

**Figure 6:**
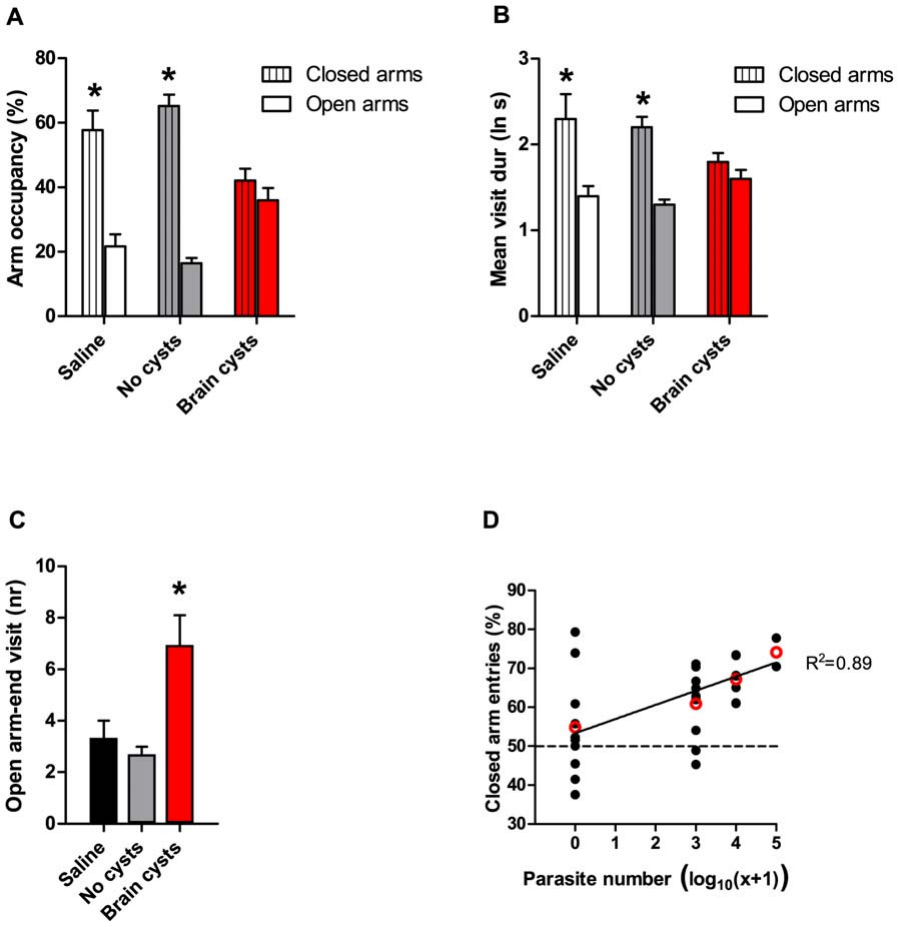
Alterations in fear/risk related behaviors in the elevated plus maze in mice with brain cysts. (A) Chronically infected mice did not show the bias for closed arm *versus* open arm occupancy displayed by control groups. Occupancy of the center of the maze was the same across groups and is not represented. (B) The longer visits to closed arms observed in control groups were not detected in animals with brain cysts (ln s – natural logarithm transformation of visit duration in seconds). (C) In contrast with controls, infected animals showed a higher number of visits to the distal-most point of the open arms. (A–C) Saline group, n = 10; No cysts group, n = 20; Brain cysts group, n = 19. (D) The control group for parasite injection (n = 20) displayed a bias for closed arm entry, which increased with injected parasite load. Individual animals are represented as closed circles (black) whereas open circles (red) indicate the average values of closed arm entries (above chance) for each parasite load.

We next investigated the bias for occupancy of closed *versus* open arms in the different groups, and observed that the normal bias towards spending more time in closed arms observed in both control groups was lost in chronically infected mice (Figure 6A, *F*_(2,45)_ = 11.6, p<0.05; *post hoc* tests between arms in Brain cysts group, p>0.05). Furthermore, the duration of visits to each arm was also affected, with control animals showing longer visits to closed rather than open arms (Figure 6B, Saline t(9) = 2.62; No-cysts t(19) = 6.47, p<0.05), and animals with cysts performing visits of similar duration to both closed and open arms [t(18) = 1.26, ns, p = 0.22]. We also scored the number of visits to the very end of the open arms (a behavior that could be regarded as highly fearful or risky) and observed that chronically infected animals performed more of these visits when compared with the infection control group (Figure 6C, *H*_(2)_ = 11.3, corrected for multiple comparisons p<0.02). Taken together, these results indicate that chronic *Toxoplasma* infection has profound effects on risky behavior/unconditioned fear responses. Animals with brain cysts covered longer distances in the exposed arms and performed shorter and faster visits to closed arms. Furthermore, these animals showed a loss of bias towards the occupation of the safer space (moving faster in the closed arms) and an increase in the number of visits to the more exposed region of the open arms, which is consistent with risky behavior.

Interestingly, mere exposure to the parasite, without chronic infection and development of brain cysts, also had effects on animal behavior. We detected a bias towards entering the safe areas in the No-cysts group: these animals displayed an increase in the percentage of closed *versus* open arm entries (50% being the chance probability value), and this increase was correlated with the number of parasites injected (Figure 6D, R^2^ = 0.89, *F_(1)_* = 17.8, p = 0.05).

### Behavioral alterations cluster into different categories

In order to identify the specific contributions of the different variables measured above to the observed behavior in all experimental groups, we performed principal component analysis (PCA) on the scores of each animal in each of the behavioral measures. This analysis resulted in the extraction of five factors which classified and described the observed behavioral modifications and accounted for 85% of the total variance. Factor loadings obtained after Varimax rotation (see Materials and Methods) are shown in Table 1. Factor 1 explained 23% of the variance and the significant variables that were positively loaded in this factor constituted common components of mouse behavior in both the open field and the elevated plus maze. Given that these variables represent different aspects of horizontal movement, this factor likely reflects general locomotion. Factor 2 accounted for 18% of the variance. The variables that were positively loaded in this factor corresponded to behaviors in the center zone of the open field whereas the negative loadings corresponded to general locomotion variables, suggesting that this factor accounted for behavior in the exposed zone of the open field test (center). Factor 3 explained 17% of the variance and was positively loaded by variables that described behaviors in the non-exposed zone (border) of the open field. Factor 4 accounted for 14% of the variance and variables related to characteristics/structure of general locomotion and vertical movement in the open field loaded positively on this factor, whereas variables that described the absence of horizontal movement loaded negatively. This indicated that this factor may reflect the structure of exploratory behavior. Finally, factor 5 accounted for 13% of the variance. This factor was positively loaded mostly by variables that described features measured in the elevated plus maze test, and more specifically in the open arms. Therefore, we interpreted this factor as representing changes in risk/unconditioned fear behavior.

**Table 1:** Factors obtained from principal component analysis of behavioral variables measured in the open field (OF) and elevated plus maze (EPM).

	Factor				
	1	2	3	4	5
OF - Overall Speed	0.807			0.402	
EPM - Overall speed	0.661				0.454
OF - Speed *versus* time (1st sec)	0.849				
OF - Speed border zone	0.829			0.415	
OF - Speed center zone	0.779	−0.466			
EPM - Overall distance travelled	0.682				0.525
OF - Overall distance travelled	0.752			0.449	
OF - Dist. travelled border zone	0.769	−0.418			
OF - Dist. travelled center zone	0.501		−0.465	0.599	
OF - Bout duration	0.507			0.650	
OF - Nr entries center zone	0.600		−0.556	0.408	
EPM - Open arm end visit	0.588				0.741
OF - Freezing epis. center zone		0.909			
OF - Freezing time center zone		0.831			
OF - Time immobile center zone		0.792		−0.460	
OF - Non-loc periods center zone		0.756		−0.447	
OF - Visit duration center zone		0.844			
OF - Occupancy center zone		0.805	−0.452		
OF - Overall time freezing			0.949		
OF - Freezing epis. border zone			0.943		
OF - Freezing episodes border			0.917		
OF - Time immobile border zone			0.778	−0.419	
OF - Visit duration border zone			0.619		
OF - Non-loc periods border				−0.791	
OF - Bout nr				−0.771	
OF – Rearings				0.411	0.450
EPM - Distance travelled open arms					0.782
EPM - Visit duration open arms					0.859
EPM – Occupancy open arms					0.904
*Variance explained (%)*	23.000	18.000	17.000	14.000	13.000
*Variance explained (Cumulative %)*	85.000				

**Figure 7:**
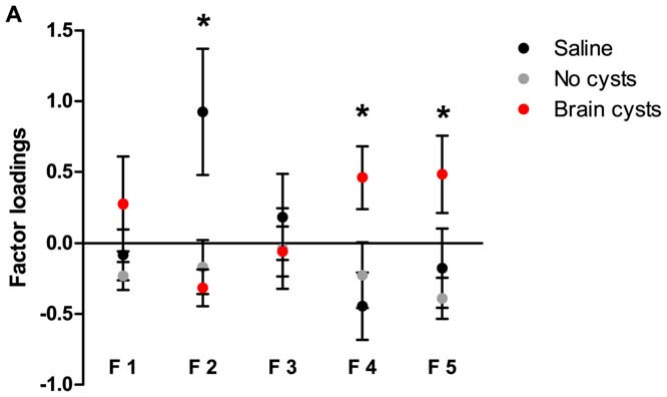
Factorial scores derived from principal component analysis of open field and elevated plus maze variables. Factor scores were calculated for individual animals in the different experimental groups using loadings derived from PCA. Animals with brain cysts showed different loadings from control groups for Factors 4 (F4) and 5 (F5). Factor 2 (F2) loadings were different in cyst-containing mice compared with saline injected animals, but similar to infection control mice with no cysts.

Next, we analyzed how each animal in the different experimental groups scored for the individual five factors that were extracted (Figure 7). This analysis demonstrated that the first component (F1), likely reflecting general locomotion, was fairly similar between the different experimental groups (*H*_(2)_ = 0.67, ns, p = 0.71). However, cyst-containing mice did exhibit a marked distinction in the structure of their behavior (F4, *H_(2)_* = 6.74, corrected for multiple comparisons p<0.02). Interestingly, animals with brain cysts also showed significantly different loadings for behavior in the exposed zone compared with saline animals (F2, *H_(2)_* = 7.45, corrected for multiple comparisons p<0.02); however this factor did not discriminate between animals with brain cysts and No-cysts control group (p>0.05). This result further demonstrated that exposure to parasites without the accumulation of cysts in the brain was sufficient to modify behavioral responses in the center area. Behavior in the border zone of the open field was similar between groups (F3, *H*_(2)_ = 1.21, ns, p = 0.54). Finally, the behavior component related to risk/unconditioned fear also discriminated cyst-containing animals from control groups (F5, *H_(2)_* = 7.09, corrected for multiple comparisons p<0.02). These results suggest that the alterations in behavior observed in animals with brain cysts mostly reflect changes in the structure of exploratory behavior and risk/unconditioned fear.

### *Toxoplasma* cysts show non-random distribution in mouse brains

**Figure 8:**
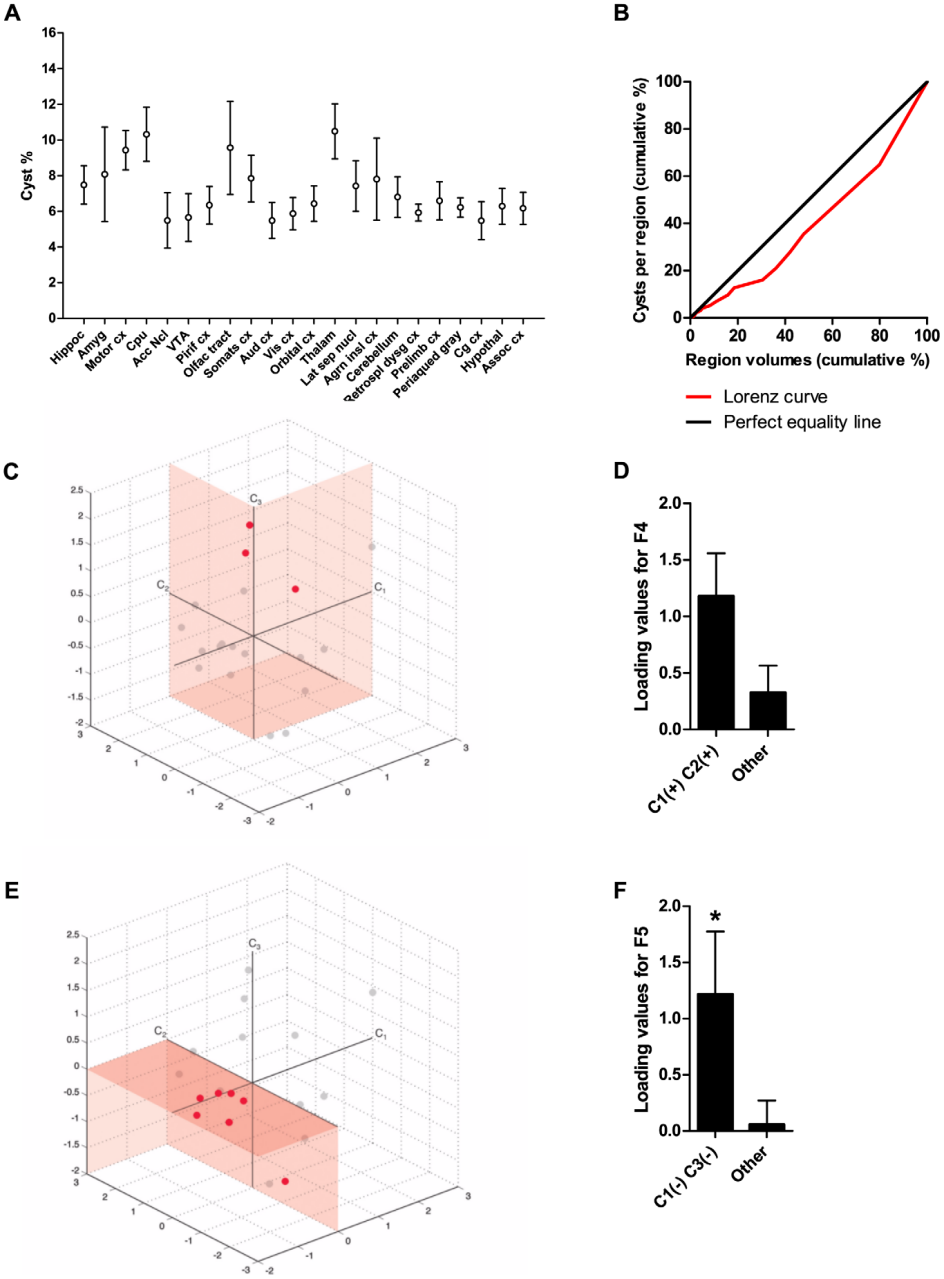
Non-random distribution of *Toxoplasma* cysts in mouse brains. (A) Cyst frequency and distribution in different mouse brain areas (n = 23). (B) Lorenz curve of cumulative cyst presence in relation to relative brain volume of specific areas. Cyst distribution deviated from perfect equality and was therefore not random. (C) Representation of the quadrant intersection [C1(+) C2(+)] of the cyst localization PCA space (shaded area). Animals with loadings in this quadrant (red dots) were not different from the rest of the animals with brain cysts, for behavioral component F4. (D) Infected animals which co-clustered in the [C1(+) C2(+)] quadrant of the cyst localization PCA space (n = 3) did not show significantly higher factor loadings for behavioral component 4 (F4) than animals that did not cluster in this quadrant (n = 16). (E) Representation of the quadrant intersection [C1(−) C3(−)] of the cyst localization PCA space (shaded area). Animals with high loading values for behavioral component F5 cluster in this quadrant (red dots). (F) Infected animals which co-clustered in the C1(−) C3(−) quadrant of the cyst localization PCA space (n = 7) showed significantly higher factor loadings for behavioral component 5 (F5) than animals that did not cluster in this quadrant (n = 12). Hippoc, hippocampus; Amyg, amygdala; Motor cx, motor cortex; Cpu, caudate putamen; Acc Ncl, accumbens nucleus; VTA, ventral tegmental area; Pirif cx, piriform cortex; Olfac tract, olfactory tract; Somats cx, somatosensory cortex; Aud cx, auditory cortex; Vis cx, visual cortex; Orbital cx, orbital cortex; Thalam, thalamus; Lat sep nucl, lateral septal nucleus; Agrn insl cx, agranular insular cortex; Retrospl dysg cx, retrosplenial dysgranular cortex; Prelimb cx, prelimbic cortex; Periaqued gray, periaqueductal gray; Cg cx, cingulate cortex; Hypothal, hypothalamus; Assoc cx, association cortex.

Given these effects of chronic *Toxoplasma* infection in unconditioned mouse behavior, we performed a detailed analysis of the distribution of cysts across the whole brain of infected animals. Analysis of cyst frequency and brain areas in which they occurred demonstrated that cysts were distributed across several regions (Figure 8A), which have been described to control different functions such as sensory processing, movement, spatial memory, learning, anxiety and defensive behavior. Interestingly, we observed no increased cyst accumulation in amygdalar structures [8], classically associated with the modulation of innate fear, relative to other structures (no overall difference between areas, *H*_(21)_ = 26.5, ns, p = 0.19).

To determine whether the presence of cysts in specific regions was uniform, i.e. related to the relative volume of each region in the brain, we used a Lorenz curve (Figure 8B). The diagonal represented perfect equality in cyst distribution and deviations from this perfect line correspond to a selective distribution favoring some regions. The greater the deviation from this perfect line, the bigger the inequality of distribution. This analysis showed that there was substantial deviation in the distribution of cysts, and therefore cyst distribution was not random. Next, we performed PCA to determine which cyst-containing areas clustered together. Three factors were extracted for the localization of cysts in the brain, which accounted for 51% of the total variance. Factor loadings obtained with variable principal normalization are shown in Table 2. Factor 1 explained 25% of the variance and the higher factor loadings in this component corresponded to several brain areas involved in sensory processing, motor control, decision-making, spatial navigation, defensive behaviors, homeostasis, and fear/emotion. Factor 2 accounted for 14% of the variance and area variables related to sensorimotor integration and alertness, olfaction and value processing were loaded positively, while areas related to spatial navigation and movement were loaded negatively. Factor 3 explained 12% of the variance and clustered variables involved in movement, motor control, homeostasis, reward, and olfaction. Overall, this analysis showed that even though brain areas were clustered into distinct categories, these were not easily grouped by a common function or spatial localization within the brain (for example, cortical *versus* subcortical areas).

**Table 2:** Factors obtained from principal component analysis of cyst distribution across brain areas.

	Factor		
	1	2	3
Insular cortex	0.487		0.470
Somatosensory cortex	0.785		
Hypothalamus	0.578		0.493
Hippocampus	0.440	−0.523	
Caudate putamen	0.513	−0.548	
Orbital cortex	0.558	0.535	
Motor cortex	0.784		
Association cortex	0.628		
Cingulate cortex	0.611		
Amygdala	0.533		
Lateral septal nucleus	0.626		
Claustrum	0.500	−0.675	
Reticular nuclei	0.441	0.654	
Thalamic nucleus	0.451		
Accumbens nucleus	0.500		0.561
Prelimbic cortex	−0.473		0.465
Tenia tecta		0.634	
Olfactory tract			0.475
Cerebellum			−0.481
VTA			−0.585
*Variance explained (%)*	25.000	14.000	12.000
*Variance explained (cumulative %)*	51.000		

### Behavioral modifications correlate with particular combinations of cyst distribution

The fact that cyst distribution was not random suggested that the behavioral modifications detected in chronically infected mice resulted from cyst presence in specific brain areas. To address this question, we performed a Pearson correlation analysis between individual behavior and cyst localization components. No significant correlations were detected between any behavioral component and cyst localization component (data not shown).

Since no correlation between behavior and cyst localization components was observed using pair-wise comparisons, we investigated whether particular combinations of cyst localization components could account for specific behavioral changes. This was equivalent to determining whether animals which co-clustered in intersections of the different quadrants of the cyst-localization PCA-space showed different behavior from animals that did not co-cluster in that quadrant (Figure 8C–F). We discovered that animals that co-clustered in a particular area of the cyst-containing space [(C1(−) C3(−)] had higher loadings for behavioral component F5 (Figure 8E,F, t(17) = 2.30, p<0.05) than animals that did not co-cluster in this quadrant. These analyses suggested that there was a particular combination of cyst localizations in the brain that biased mice towards increased risk behavior (described by component F5). Although a similar tendency was uncovered for the structure of exploratory behavior described by factor F4 (Figure 8C,D), this bias was not significant (t(17) = 1.46, ns, p = 0.16).

Taken together, these results showed that the changes in the structure of exploratory behavior and risk/unconditioned fear observed in chronically infected animals depended on cyst presence, but were only partially related to the spatial distribution of cysts in the brain.

## Discussion

We investigated the behavioral changes in *Toxoplasma*-infected mice that developed brain cysts in two environments with exposed *versus* non-exposed areas and observed that chronic infection modifies a series of specific aspects of unconditioned behaviors. In infected animals, exploratory locomotion was more common, faster and had higher initial acceleration. Strikingly, this locomotion was also organized differently relative to control groups: infected animals exhibited fewer and longer segments of ambulatory movement. Additionally, these animals did not display normal cautious behaviors when placed in a novel environment, and they also showed differential responses to unsafe areas with reduced unconditioned fear and more risky behaviors.

When we reduced the dimensionality of 37 behavioral features to 5 different factors using principal component analysis, we were able to cluster the behavioral differences between animals with brain cysts and the different control groups into two main factors. These factors mostly represented features related to the microstructure of exploratory behavior (F4) and risk/unconditioned fear (F5). Surprisingly, no significant differences were observed between experimental groups for the factor representing features of general locomotion (F1). This result indicates that the behavioral modifications in exploratory locomotion observed in mice with brain cysts likely reflected the altered structure of exploratory behavior, rather than representing hyperactivity.

The data presented here suggest that chronically infected animals show differential responses to exposed areas, away from normal defensive behavior, and are consistent with previous studies in infected rats [6]. However, our data do not allow us to discriminate whether these behavioral alterations are caused by changes in the way environmental risk is evaluated, and/or by changes in producing/organizing the appropriate behavioral responses.

We observed that although cysts in chronically infected animals were not randomly distributed across different brain areas, there was no special frequency of cyst accumulation in specific brain regions (e.g. in the amygdala as has been reported in previous studies [8]). However, we found evidence that particular combinations of cyst distribution in the brain biased the animals for specific behavioral phenotypes (changes in risk/unconditioned fear, described by F5). These results suggest that cyst accumulation in different areas of a particular circuit may lead to similar behavioral alterations and thus that the parasite may have experienced selective pressure to manipulate functional neuronal circuits rather than a specific area.

Another possibility is that the parasite would change the secretion or function of general neuromodulators. For example, altered concentrations of catecholamines and indolamines have been observed in whole brain extracts of *Toxoplasma*-infected mice. In particular, dopamine levels have been reported to be higher in *Toxoplasma*-infected mouse brains [19], and a recent study showed that brain cysts were able to produce tyrosine hydroxylase, an enzyme involved in dopamine biosynthesis, and also that *Toxoplasma*-infected dopaminergic neurons showed an increase in dopamine synthesis and release [20]. These observations suggest that dopamine could be involved in some of the behavioral modifications described here, namely in movement structure (F4), since these seem to be less dependent on cyst localization.

An interesting, and to our knowledge, previously unreported phenotype was observed in the infection control group. In this group, systemic parasitic contact did not result in clinical symptoms of infection (as indicated by lack of antibodies against *Toxoplasma*, weight loss and brain cysts). The absence of detectable circulating antibodies against this parasite is intriguing. One possibility would be that the concentration of antibodies is below the detection levels of the method used and, alternatively, that mice in this experimental group used cell-mediated immune response mechanisms. In fact, cell-mediated immune reactions (involving CD4^+^ and CD8^+^ T cells and macrophages) are believed to be involved in the defense against intracellular parasites (reviewed in [21]). Furthermore, there are cell-autonomous defense mechanisms implicated in resistance to *Toxoplasma* infection, involving IFN-inducible immunity-related GTPases, which drive targeted destruction of parasite-containing vacuoles (reviewed in [22]). Despite the absence of infection symptoms and brain cysts in this experimental group, contact with the parasite was sufficient to alter behavioral responses to exposed areas. This is consistent with previous studies showing that asymptomatic *Campylobacter jejuni*-infected animals display an increase in closed arm entries in the elevated plus maze [23]. Subsequent studies have demonstrated that peripheral infection, even in the absence of a measurable immune response, can activate viscerosensory pathways that interface with defensive brain networks [24]. This suggests that in our infection control animals, information about transient *Toxoplasma* infection could have been relayed to visceral sensory structures in the brain therefore leading to the observed behavioral changes. It is also of interest that the behavioral changes observed in this experimental group are generally opposite to those observed in animals with brain cysts. This further suggests that the behavioral alterations in the different groups arise by different mechanisms. In the “Brain cysts” group the behavioral modifications observed are related to cyst presence in the brain (and specific cyst localizations in the case of F5), whereas in the “No-cysts” group alterations may result from a systemic effect that produces changes in the brain.

The behavioral differences described here for infected animals are not easily explained by general debilitation due to *Toxoplasma* infection, because animals with brain cysts show increased performance in some behavioral variables and decreased performance in others. Rather, these results suggest that chronically infected mice interact with their environment differently than non-infected animals. This change results in maladaptive behaviors, such as longer bouts of locomotion in exposed areas, or increased exploration of unsafe zones. These behaviors would render infected animals more vulnerable to predation or environmental risks, the latter resulting in enhanced capture probability. This is unlikely to be a general effect of parasite-altered behavior. Parasite-driven behavior modifications do not necessarily lead to increases in trappability or general predation, since other studies have shown that parasites can manipulate intermediate host behavior to actually decrease general predation (reviewed in [25]).

One interesting hypothesis is that in addition to the selective pressure to increase the vulnerability of infected rodents to felid hosts (where sexual and asexual reproduction occur), there may have been selective pressures to increase transmission to host predators in general (including intermediate hosts, where asexual reproduction occurs). Several studies have suggested a number of mechanisms through which *Toxoplasma* could increase the likelihood of predation by the definitive host (reviewed in [26]). The behavioral modifications described here could serve to increase the overall capture probability observed in *Toxoplasma*-infected rodents [11], and thus be of evolutionary importance for parasite transmission between a variety of different hosts. Even though there is no consensus over the actual contribution of asexual reproduction to *Toxoplasma* population structure (reviewed in [27]), some studies suggest that *Toxoplasma* has been expanding largely clonally (asexually) for the past 10 000 years, with the interesting implication that expansion of intermediate host range could be one of the driving forces behind the success of this parasite [28], [29], [30], [31]. Some studies even argue that there is no reason why *Toxoplasma* could not skip the definitive host altogether, using carnivorism and scavenging behaviors to move within the food chain [28]. This would imply that the parasite would also be under selective pressure to manipulate behavior in intermediate hosts other than the rodent, including those that would not be normally predated by felids. In this respect, it is interesting to note that a series of studies have shown that chronic toxoplasmosis in humans can result in behavioral modifications, like increased activity, decreased reaction times and altered personality profiles [32], [33], [34], [35], [36].

The increase in host range and success of clonal expansion of *Toxoplasma* populations could result from the acquisition of the ability to directly infect successive intermediate hosts after cyst ingestion [31]. This bypass of sexual reproduction is absent from a parasite closely related phylogenetically to *Toxoplasma*, *Hammondia hammondi* [31], [37]. This cyst-forming parasite has a limited host range, with cats as definitive hosts and rodents as unique intermediate hosts. Interestingly, *H. hammondi* cysts are rarely found in the brain [37], even though this parasite needs to reach the definitive host to complete its life cycle. Therefore, brain cyst formation during *Toxoplasma* infection may not be the result of selective pressures to increase parasite transmission to the definitive host. Instead, it is interesting to postulate that the appearance of brain cysts is related to the increase in clonal expansion and intermediate host range observed in *Toxoplasma*, by the modification of the structure and the risk of intermediate host behavior. However, given the controversy that exists regarding the relative roles of sexual and asexual reproduction for *Toxoplasma* evolution, it is also possible that the impact of the behavioral alterations observed here is mainly to increase transmission to the definitive host. Further studies are needed to investigate these postulates.

## Materials and Methods

### Ethics statement

All procedures were reviewed and performed in accordance with the Instituto Gulbenkian de Ciência Ethics Committee guidelines, and approved by the Portuguese Veterinary General Board (Direcção Geral de Veterinária, approval ID 018831).

### Parasite and mouse strains

Female C57BL/6J (7-week old; housed four per cage; The Jackson Laboratory, Maine, USA) were used.

The *Toxoplasma gondii* strain used (ME49) was obtained from Dr. Andrea Crisanti (Department of Experimental Medicine and Biochemical Sciences, University of Perugia, Italy). This parasite strain (type II) was genetically modified to express green fluorescent protein (GFP) under the control of the cyst-stage specific BAG1 promoter, with infection dynamics similar to the parental strain [12].

### Host cells and parasite culture

Vero cells (green monkey kidney cells, CCL-81™, ATCC) were grown in Dulbecco's Modified Eagle Medium (DMEM, Gibco), supplemented with 10% newborn calf serum (FBS, Sigma), 50 000 U penicillin (Gibco), 50 mg streptomycin (Gibco) and 2 mM L-Glutamine (Sigma). Parasites were maintained as tachyzoites and propagated *in vitro* by serial passage on monolayers of Vero cells.

### Mouse infection and experimental groups

Mice were weighed and inoculated intraperitoneally with tachyzoites, using 1 ml syringes (29 G, 0.33 mm×12.7 mm). Three different parasite loads were used: 1000, 10 000 and 100 000 tachyzoites, in a final volume of 200 ul PBS (per injection), resulting in a mouse mortality rate of 14.3% and 19.2% for the 10 000 and 100 000 parasite loads, respectively. The different parasite loads resulted in similar numbers of brain cysts as shown in Figure 1E, so for most analyses all animals were combined in a single treatment group (“Brain cysts”, n = 19). Two experimental control groups were included. The first consisted of saline injected-mice (n = 10), thus controlling for injection (“Saline” group). The second was composed of parasite-injected mice (n = 20), which did not show weight loss, tested negative for antibodies anti-*Toxoplasma* (see below), and did not form parasitic cysts in the brain, thus controlling for the exposure to the parasite (“No-cysts” group). Animals were housed in groups of four and monitored weekly for general health and weight. Weight variation in Figure 1B was calculated as the difference in weight between weeks 2 and 0 over the initial weight. Experimental data corresponds to pooling of data obtained in two separate experiments (“Saline” groups, n_1_ = 6 and n_2_ = 4; “No cysts” groups, n_1_ = 15 and n_2_ = 5; “Brain cysts” groups, n_1_ = 3 and n_2_ = 16). In each experiment, the behavioral measurements were performed nine weeks post-infection.

### Brain harvesting and histology

Nine weeks post-infection, animals were sacrificed after completion of the behavioral tests. First, animals were anesthetized with isofluorane, followed by intraperitoneal injection of Ketamine/Xylaxine (∼5 mg/Kg xylazine; 100 mg/kg ketamine). Immediately before transcardial perfusion, blood was collected and serum samples analyzed for the presence of anti-*Toxoplasma* antibodies using the latex agglutination assay Toxocell (Biokit). Animals were then perfused with saline and 4% paraformaldehyde, and brains extracted for histological analysis. Brains were cryoprotected by overnight immersion in saline containing 30% sucrose (Sigma) and each brain was sectioned coronally (40 µm slices) using a Leica cryostat CM3050S, set at −25°C. Total cyst number and localization was assessed for all brain slices.

### Immunohistochemistry

To enhance the fluorescence signal present in brain cysts, histological sections were processed for immunohistochemistry as follows. After washing with PBS, sections were incubated in 0.1 M Glycine in PBS, for 10 min at room temperature. The tissue sections were then permeabilized with 0.5% Triton X-100 in PBS, for 10 min at room temperature. After a blocking step (incubation for 30 min, at room temperature with 10% FBS:0.2% Triton X-100:PBS), sections were incubated, for 1 h at room temperature, with the antibody against GFP conjugated with the Alexa 488 fluorochrome (1∶500, rabbit polyclonal, Molecular Probes). Sections were then washed and DNA was counterstained with 4′,6-diamidino-2-phenylindole (DAPI, Sigma).

After mounting and sealing of sections, these were visualized using a wide field fluorescence microscope (AxioImager, Zeiss) and a fluorescence stereoscope (Stereo Lumar, Zeiss). Images were acquired using Hamamatsu digital cameras (ORCA-ER and C8484 models).

The efficiency of infection, for each parasite load injected (Figure 1D), was calculated as the percentage of animals that contained brain cysts at 9 weeks post-injection. The percentage of cysts in a given area (Figure 8A) was calculated relative to the total number of cysts, in each animal.

### Behavioral data

Animals were housed under a 12 h light-dark cycle (lights on at 8 a.m.) with food and water available *ad libitum*. All behavioral tests took place between 8 a.m. and 1 p.m. To eliminate odor cues, all testing equipment was thoroughly cleaned between animals with a 10% ethanol solution.

Animals were first tested on the open field, and 24 h later on the elevated plus maze test.

Open field - Exploratory locomotion was tested in a white floor/black wall acrylic square arena (39.5×39.5×17.5 cm). The apparatus was placed in an enclosed area previously covered in black cloth to avoid distracting visual cues. Testing was conducted after cleaning the arena with 10% ethanol solution. Each animal was placed in the center of the arena and allowed to freely explore for a period of 10 min. The center zone was defined as 50% of the available open field area. Movement and spatial distribution of exploration was video captured (Sony Handycam DCR-SR57E, 25 frames/second).

Elevated plus maze - This apparatus was made of black (walls) and white (floor) acrylic and consisted of four arms (two open and two enclosed by 15 cm high walls), 39.5 cm long and 5 cm wide. Each arm of the maze was attached to a supporting structure and elevated 40 cm off the floor. Similar levels of illumination were present in both open and closed arms. The arm surfaces and closed sides were wiped clean with 10% ethanol and allowed to air dry. Mice were placed at the junction of the four arms of the maze, facing an open arm and allowed to explore the maze for 5 min. Movement was recorded using a video system (Sony Handycam DCR-SR57E, 25 frames/second).

### Behavioral data analysis

Video behavioral data was scored automatically using the Anymaze software (Stoelting, USA). Graphics were elaborated using Graphpad Prism (GraphPad Software, Inc., USA). The speed over time plot in Figure 2C was based on distance travelled per second, in the first 4 seconds of movement segments ranging 6–15 s. The initial acceleration during the first second of a movement bout was calculated as the slope of the one second line in Figure 2C. The latency plot in Figure 2D corresponds to the time elapsed until animals initiate locomotion. The coefficient of variation in Figure 2F was calculated as the standard deviation divided by the average distance travelled over time for each animal.

Movement bouts in Figure 3A were scored as the number of horizontal (planar) transitions from immobility to mobility and back. Bout frequency (Figure 3C,D) was calculated as the percentage of movement bouts of specific durations (e.g. 0 to 5 s). Center occupancy in the open field test (Figure 4A) corresponds to the percentage of time spent in the center zone. Relative non-locomoting periods (Figure 4D) were scored as the percentage of episodes of no horizontal movement per specific apparatus area (center, border). In this analysis, immobility sensitivity was set at 70%, which is the percentage of the animal that needs to remain in place for 2 s for it to be scored as immobile/non-locomoting. Freezing was calculated as the percentage of time the animal spent freezing (absence of motion apart for respiratory-related movements) relative to time spent in a specific area (Figure 4F). Rearings (Figure 4G) were scored as the number of rearings against the open field walls relative to time spent in the border zone. Center occupancy in the elevated plus maze was not represented in Fig. 6A because there were no significant differences between experimental groups. Arm occupancy (Figure 6A) was calculated as the percentage of time spent in each arm type.

Data transformation using natural logarithm operation was applied to the mean visit duration data (seconds, Figure 6B) to achieve normality. In Figure 6D, a variable transformation was performed [log_10_(x+1) on parasite loads (0, 1000, 10 000 and 100 000 parasites)] to meet the variable linearity condition necessary for regression analysis.

### Statistical analysis

Data are displayed as mean ± SEM. All statistical analyses were carried out using SPSS statistics 17 software (IBM, New York). Data distribution was first tested for normality (using the Kolmogorov-Smirnov test) and for variance homogeneity (Levene's test). Data not violating normality was analyzed using paired *t*-tests, independent samples tests, one-way ANOVA or two-way mixed design ANOVA, followed by the Bonferroni *post hoc* test. For data not conforming to homogeneity of variance, Welch correction was used for the ANOVA analysis and Games-Howell correction for *post hoc* comparisons. Data that did not conform to normality was analyzed using the Kruskal-Wallis test and Mann-Whitney U-test with Bonferroni correction. Differences were considered significant at p<0.05 and were indicated in the figures with an asterisk. In order to identify any underlying structure and possible interrelations in the set of variables/behaviors studied, we used multivariate factor analysis (MFA) to reduce data dimension, while retaining as much of the original information as possible.

Principal Component Analysis (PCA) with orthogonal (independent) rotation (Varimax) was used so that the variance within each extracted factor was maximized to allow for an easier interpretation of the factor structure. The appropriateness of this analysis was assessed by the Kaiser-Meyer-Olkin measure of sampling adequacy test (KMO) and the Bartlett's sphericity test. Inclusion of any given variable required an absence of any strikingly aberrant values that could affect the robustness of this analysis. The interpretable factors identified (loadings >0.40 were considered relevant for a specific factor) represent the primary parameters or latent variables that are being measured. An equivalent data matrix was generated as Anderson-Rubin factor scores. These scores represent each animal classification on the identified factors with means of zero and standard deviations of one (z-scores), with individual scores for each identified factor. *Factor analysis of behavioral variables for all experimental groups:* MFA with a Varimax (orthogonal) rotation of 29 out of 37 features from open field and elevated plus maze behavioral experiments was performed with data obtained from 48 animals (10 saline, 19 with No-cysts and 19 with cysts). An examination of the KMO measure of sampling adequacy suggested that the sample was factorable (KMO = 0.716). Five factors were extracted with Eigen values higher than 1 accounting for 85% of all variance.

Categorical Principal Components Analysis (CATPCA) was used for multivariate factor analysis of the data relative to cyst distribution across the brain. This method was selected given that data was sparse, which impaired the use of standard statistical procedures, and was performed using variable principal normalization. As in the previous case, this is a dimensionality reduction of a set of variables while accounting for as much of the variation as possible. Scale values are assigned to each category of every area variable so that these values are optimal relative to the principal components' solution. Variables in the analysis were assigned component scores based on the quantified data. *Factor analysis of brain cyst area variables:* CATPCA was performed on 20 out of 80 area variables in 19 animals (only areas containing cysts in more than 25% of the animals were considered, given that this type of analysis is more robust when the majority of variables are not scored as zero). The analysis produced a three-factor solution accounting for 51% of all variance. *Correlation analysis between behavior and brain cyst area variables:* To determine the relationship between CATPCA extracted factors and the behavior PCA factors we performed a factor correlation analysis (Pearson coefficients).

To determine whether cyst presence in specific brain regions was connected to the relative volume occupied by these regions, a Lorenz curve was calculated. First, the number of cysts detected was ordered lowest to highest and on the horizontal axis the corresponding relative volumes of specific brain regions (cumulative percentage using data described in ref. [38]) was plotted (some areas were combined for this analysis; for example, cortical areas were collapsed and collectively designated as neocortex). On the vertical axis, the cumulative percentage of the number of cysts per region was represented. The diagonal represents perfect equality in cyst distribution; for example, the bottom 10% of region volume would contain 10% of the cysts detected. Deviations from this perfect line represent a selective distribution favoring some regions. The greater the deviation from this perfect line, the bigger the inequality of distribution. The different regions considered are shown in Table 3.

**Table 3:** List of brain areas considered in the Lorenz curve analysis and respective brain volumes in mice, according to reference [38].

	Average vol (mm^3^)
Inferior colliculi	5.7
Fimbria	2.6
Internal capsule	2.6
Superior colliculi	8.6
Globus pallidus	3.2
CC/External capsule	14.8
Amygdala	11.6
Olfactory bulb	22.9
Hypothalamus	11.8
Cerebellum	54.2
Hippocampus	25.7
Thalamus	26.8
Caudate putamen	26.6
Neocortex	144.9
Rest of the brain	91

## Supporting Information

Video S1 **Spontaneous exploratory locomotion of a saline injected mouse in the open field test.** The animal explores the environment.
(WMV)

Video S2 **Spontaneous exploratory locomotion of a cyst-containing mouse in the open field test.** The animal moves more and faster than saline controls.
(WMV)

Video S3 **Saline control: Latency to move after placement in the center of the open field.** The animal takes some time to initiate movement.
(WMV)

Video S4 **Cyst-containing animal: Latency to move after placement in the center of the open field.** The mouse moves immediately after being placed in the exposed area.
(WMV)

Video S5 **Fear-related responses in a saline injected animal in the elevated plus maze test.** The animal displays a very cautious approach to the open arms and does not spend a lot of time exploring these exposed areas.
(WMV)

Video S6 **Fear-related responses in a cyst-containing animal in the elevated plus maze test.** The mouse explores the exposed areas/open arms abundantly.
(WMV)
